# Rheology in Polymer Processing: Modeling and Simulation, Krzysztof Wilczyński, Hanser Publishers, 1st Edition. 2020, ebook ISBN: 978-1-56990-661-3; Print ISBN: 978-1-56990-660-6; 390 Pages

**DOI:** 10.3390/polym13121981

**Published:** 2021-06-17

**Authors:** John Vlachopoulos

**Affiliations:** Department of Chemical Engineering, McMaster University, Hamilton, ON L8S 4L7, Canada; vlachopj@mcmaster.ca

In 1945, about one million tons of polymer resins were produced globally, and due to rapid growth, 400 million tons were produced in 2020. The conversion of polymer resins to useful plastic products is accomplished mainly through extrusion, injection molding, blow molding, thermoforming, calendering and compression molding; collectively referred to as polymer processing. The growth of the industry is due to food packaging, transportation (mostly light weighting of automobiles), building/construction, electric/electronic, textiles, medical products, and other sectors of the economy. The global market size is about 1.2 trillion US dollars per year, with roughly $600 billion being the cost of raw materials and $600 billion the cost for processing. Demands for the improvement of properties, the increase in production rates, and the reduction in costs, have led to the intensification of research, development, and education efforts in polymer processing around the world.

The first chapter in Professor Wilczynski’s book is devoted to rheological fundamentals, including an introduction to continuum mechanics, viscosity of polymers, models for viscosity, concepts of viscoelasticity, and viscoelastic models. It is a pedagogically excellent introduction to some very difficult concepts. It is mathematically rigorous, but also easy to follow.

The second chapter describes both the theoretical and practical aspects of rheometry, including potential errors, slip effects, extensional viscosity, and normal stresses. It covers all methods of measurement for obtaining the experimental data required as inputs in process simulation software.

The third chapter provides an overview of the various polymer processing operations and includes very informative graphics which show how plastics production machinery works. This chapter is of great value to students without any background in polymer rheology and processing.

The fourth chapter focuses on process modeling and includes careful step-by-step derivations of the most important equations, which describe flow through the most frequently encountered types of channels.

The fifth chapter is devoted to the modeling of extrusion and includes detailed derivations of models for solids transport, melting in extruders, and flow through extrusion dies. With the step-by-step approach of this book, it is easy to understand how the various models are developed. It provides useful insights into the enormous challenges in building models for the prediction of the performance of extrusion machinery.

The sixth chapter is on computer modeling for polymer processing. It includes several examples with actual data and colorful computer visuals. The reader will be able to quickly appreciate what is needed for the flow simulation of polymer melt flows though extruders, dies and molds, and what sort of results will be obtained. It is of great value to students embarking on a new research project for an advanced university degree, and to industry professionals considering an investment of money and effort in computational tools for improving plant operations.

Instructors and students will find this book very useful. Instructors will have a pedagogically excellent sequence of lecturing material for presentations. They will certainly appreciate the carefully selected and presented topics, whether it is rheology, rheometry, modeling or simulation. Students will be able to follow the development of concepts, methodologies, and models without much previous background knowledge. This book will also be useful to practicing polymer processing professionals. For example, most people working in the plastics industry have not had special education in polymer rheology or processing. They usually have degrees in mechanical or chemical engineering, and they have had on-the-job training with trial-and-error procedures. They may have been able to run and to improve processing or even to innovate, but with the help of the carefully selected and organized knowledge presented in this book, they will be able to gain useful insights into the thermomechanical operations involved, and eventually they will be able to do more with less effort.

